# Case report: Dramatic response to alectinib in a lung adenosquamous carcinoma patient harbouring a novel CPE-ALK fusion

**DOI:** 10.3389/fonc.2022.998545

**Published:** 2022-12-01

**Authors:** Yanyan Qin, Fei Li, Yuan Tan, Qianqian Duan, Qin Zhang

**Affiliations:** ^1^ Department of Respiratory and Critical Care Medicine, Shanxi Provincial People’s Hospital, Shanxi, China; ^2^ The Medical Department, Jiangsu Simcere Diagnostics Co., Ltd, Nanjing, China; ^3^ Nanjing Simcere Medical Laboratory Science Co., Ltd, Nanjing, China; ^4^ The State Key Lab of Translational Medicine and Innovative Drug Development, Jiangsu Simcere Diagnostics Co., Ltd, Nanjing, China

**Keywords:** lung adenosquamous carcinoma, NGS, CPE-ALK, alectinib, IHC

## Abstract

Lung Adenosquamous carcinoma (ASC) is a rare histological subtype of lung cancer accounting for 0.4%–4% of all lung cancers. ASC is generally considered to be an aggressive cancer with poor prognosis. There is no specific standard treatment for ASC, and current treatment of ASC is relied on the guideline for non-small cell lung cancer (NSCLC). To date, only sporadic canonical EML4-ALK fusions have been reported in ASC patients, and the efficiency of ALK-TKI is still unclear in non-canonical ALK fusion positive ASC patients. Here we describe the case of a stage IV ASC patient harboring a novel CPE-ALK fusion detected *via* 74 genes panel analysis. Interestingly, the TP53 was wild-type and no another somatic mutation was found within 74 genes. In addition, immunohistochemical staining (IHC) also supports an oncogenic role for the CPE-ALK fusion. Based on these findings, the patient received alectinib 600 mg twice daily. After 4 months on treatment the patients achieved a radiological partial response (PR) and his symptoms were significantly relieved. Imaging showed that lesions of the patient were reduced, and the clinical evaluation was partial response (PR). To the best of our knowledge, this is the first report of a dramatic tumor response to alectinib in a patient with ASC harboring a CPE-ALK fusion. In addition, targeted NGS analysis may improve detection of ALK fusion in routine practice.

## Introduction

Adenosquamous carcinoma (ASC), a rare biphasic malignancy, consists of two morphologically distinct components, including Lung adenocarcinoma (LUAD) and squamous cell carcinoma (LUSC) (1). Although ASC has worse prognosis than LUAD and LUSC, the standard treatment for ASC is currently not well defined, and therapeutic decisions are made according to the treatment guidelines of non-small cell lung cancer (NSCLC) (2). With the development of precision medicine, targeted therapies can be used as first-line therapy for advanced anaplastic lymphoma kinase (ALK) rearranged NSCLC, while there are limited data on the efficacy of ALK tyrosine kinase inhibitors (TKI) in ASC due to its rarity. Currently, only sporadic classical ALK fusion have been reported in ASC patients (3, 4). Although there are some ALK-positive cases and molecular profiling studies of ASC, ALK-TKI treatment for ASC patients with ALK rearrangement has been reported in only a handful of cases (5), and no non-classical ALK fusions have been reported in ASC. Here we report the case of a 71-year-old woman with stage IV ASC harboring a CPE-ALK fusion sensitive to alectinib, which highlights the importance of ALK-TKIs in ALK-positive ASC patients even in presence of non-canonical alterations.

## Case presentation

**Figure 1:**
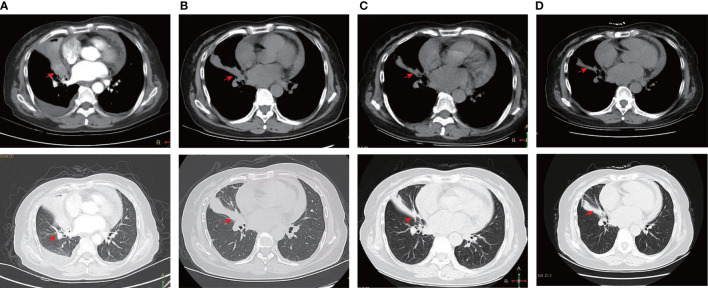
Dynamic imaging of lung lesions at different stages of treatment. **(A)** CT scan before alectnib treatment (March 1^st,^ 2022); **(B)** CT scan after two months of alectinib treatment (April 22^th,^ 2022); **(C)** CT scan after four months of alectinib treatment (June 29^th,^ 2022); **(D)** CT scan after six months of alectinib treatment (June 29th, 2022); note: the red arrow marks the location of the lesion.

**Figure 2:**
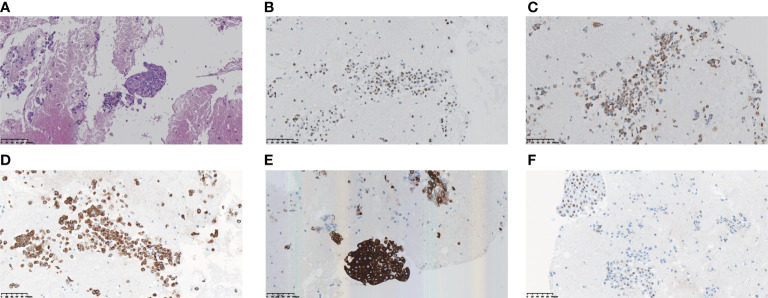
Morphology and immunohistochemistry confirmed the diagnosis of lung adenosquamous carcinoma by pathologists **(A)** H&E staining; **(B)** Positive staining of TTF-1 by immunochemistry; **(C)** Positive staining of Napsin A; **(D)** Positive staining of CK7; **(E)** Positive staining of CK5/6; **(F)** Positive staining of P63.

**Figure 3:**
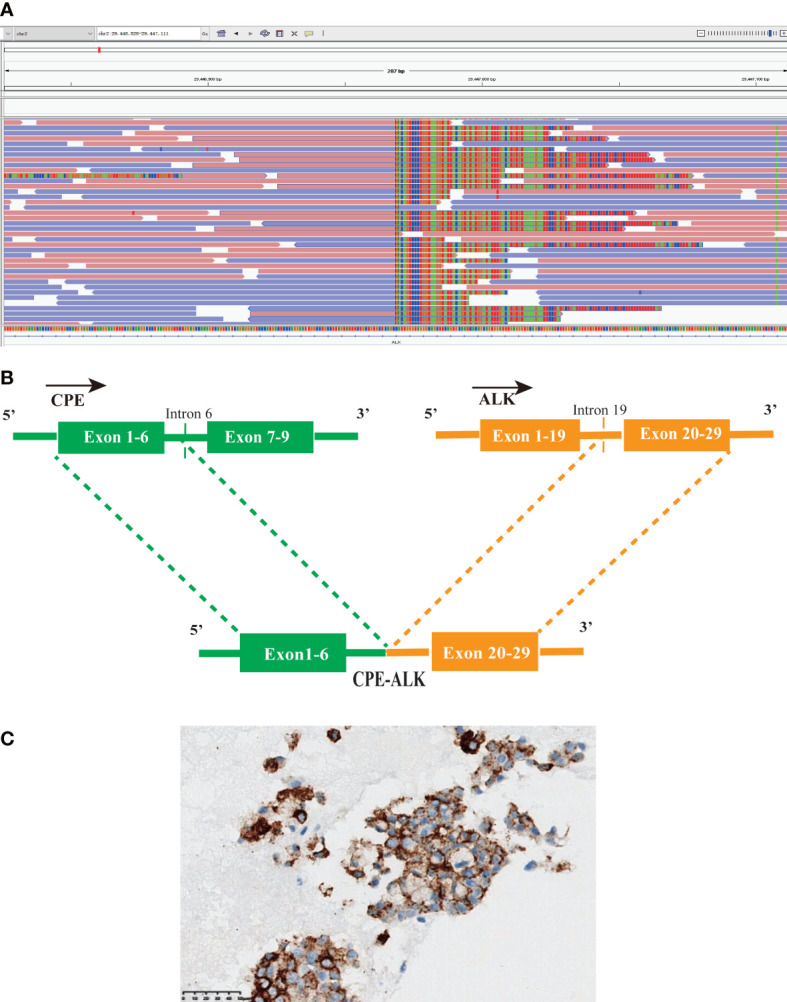
A novel CPE-ALK fusion was discovered in the ASC patient; **(A)** Sequencing reads of ALK were shown by the Integrative Genomics Viewer. **(B)** Illustration of CPE-ALK fusion. **(C)** IHC staining indicated the expression of ALK, the specimen was stained by IHC with the anti-ALK (D5F3).

A 71-year-old female was admitted to the hospital with a cough and sputum for more than two months. The patient had no history of smoking, drinking, hypertension or diabetes. A computed tomography (CT) scan showed that the middle lobe of the right lung was occupied by a mass near the hilum, with small nodules in the upper lobe of both lungs, accompanied by lymph node enlargement in area IV of the right neck (**Figure 1A**). Auxiliary examination of tumour-related markers showed the following: ferritin 180.7 ng/mL; carcinoembryonic antigen (CEA) 3.523 ng/mL; carbohydrate antigen 125 (CA125)106.341 U/mL. Lymph node puncture samples from area IV of the right neck were obtained for histopathological examination. Each slide was examined independently by two experienced pathologists based on the World Health Organization (WHO) classification criteria of lung cancer (1). The two components of adenocarcinoma and squamous cell carcinoma were both more than 10%. Pathology staining results showed TTF-1(+), Napsin A (+), CK5/6(+), CK7(+) and P63(-) (**Figure 2**). Based on pathological and imaging results, it was determined that the patient had stage IV (T1N3M1) lung adenosquamous carcinoma. Then, a 74 cancer-related gene NGS panel analysis was performed on the lymph node puncture sample by a CAP-certificated lab. The qualified DNA libraries were sequenced on Illumina NovaSeq6000 platform (Illumina, San Diego, CA) and generate 150 bp paired end reads. Base calls from Illumina NovaSeq6000 were conducted to FASTQ files. The software fastp (v.2.20.0) was used for adapter trimming and filtering of low-quality bases (6). The BWA-MEM (v.0.7.17) algorithm was performed to align to the reference genome (UCSC’s hg19 GRCh37) (7). Duplicate reads from PCR were excluded using Dedup with Error Correct. SNVs/InDels were called and annotated *via* VarDict (v.1.5.7) (8) and InterVar (9), then the variants were filtered against the common SNPs in public database including 1000 Genome Project (Aug 2015) and Exome Aggregation Consortium (ExAC) Browser28 (v.0.3). CNVs and fusions were analyzed by CNVkit (dx1.1) (10) and factera (v1.4.4) (11), respectively. A novel CPE-ALK fusion was identified (**Figure 3A**), no other molecular alterations were found among the 74 genes analyzed (gene list was shown in **Supplementary Table S1**). The results revealed that a novel fusion was generated by the 1-6 exons of CPE (carboxypeptidase E) and exons 20-29 of ALK, this fusion retains the ALK kinase domain (**Figure 3B**). IHC also supports an oncogenic protein generation of the CPE-ALK fusion (**Figure 3C**). Based on the results above, the patient was started on alectinib 600 mg twice daily. Based on the above results, the patient was started on alectinib 600 mg twice daily from March 12^th^, 2022. After approximately 1 month of alectinib treatment on April 22^th^, 2022, a CT scan showed that the lesion in the middle lobe of the right lung had decreased from 2.9 cm to 2.3 cm, that the lymph nodes had decreased from 1.8 cm to 1.2 cm, but that the lesions in the upper lobe of the right lung had remained unchanged, at 0.3 cm (**Figure 1B**). CT scan on June 29^th^, 2022, which was after four months of alectinib treatment, showed that the lesions in the middle lobe of the right lung were reduced to 1.8 cm, the lymph nodes were reduced to 1.0 cm, but the lesions in the upper lobe of the right lung were still 0.3 cm; the cough and sputum symptoms of the patient had also improved significantly. The clinical evaluation was partial response (PR) (**Figure 1C**). The patient’s condition was stable at the most recent follow-up on September 14, 2022. (**Figure 1D**). Besides that, there were no extreme drug-related side effects in this patient. Her treatment is ongoing, and we are continuing to follow up with the patient.

## Discussion

With an estimated 2.2 million new cancer cases and 1.8 million deaths, lung cancer was the second most commonly diagnosed cancer and the leading cause of cancer death in 2020 (12). ASC is defined as combining both components of adenocarcinoma and squamous cell carcinoma, with each component composing more than 10% (1). ASC has more aggressive behaviour and a worse prognosis than adenocarcinoma or squamous cell carcinoma alone (13).

Currently, there is no unified treatment for ASC, and routine care options rely on NSCLC guidelines. Treatment of lung cancer has rapidly evolved as a result of the discovery of molecular targets and recent advances in tyrosine kinase inhibitors (TKIs) against EGFR mutations and ALK fusions (14). Because EGFR mutation is the most common genomic anomaly in ASC, some studies (4, 15) have focused on the efficiency of EGFR-TKIs in EGFR-positive ASC patients and found that ASC patients had similar efficacy to EGFR TKI compared with adenocarcinoma. Despite the presence of ALK-positive cases and molecular profiling studies of ASC (2, 4, 16, 17), only a small number of ALK-TKI therapies for EML4-ALK fusion ASC patients have been reported (5), no non-classical ALK fusions have been reported in ASC. Echinoderm microtubule-associated protein-like 4 (EML4)-ALK is the canonical and most common ALK gene arrangement found in NSCLC, by which multiple EML4 breakpoints fuse in frame with the kinase domain of ALK (18). By applying NGS, over 90 ALK fusion partners have been identified in NSCLC, some ALK fusions less commonly reported in NSCLC (i.e., noncanonical ALK fusions) include kinesin family member 5B (KIF5B)-ALK, TNIP2-ALK and so on (19, 20). Although ALK-TKIs have dramatically expanded the therapeutic landscape of ALK-positive NSCLC, it remains controversial whether patients with noncanonical ALK rearrangements benefit from targeted therapy as much as those with typical ALK rearrangements. For instance, the conclusions of two studies of survival analysis between patients with classical and nonclassical fusions are contradictory (21, 22). The substantial question, of whether noncanonical fusions can unequivocally produce the corresponding transcripts or response to ALK-TKI, is still uncertain (23). Based on the ALK rearrangements found by the DNA assay, plenty of the breakpoints for ALK are in intron 19 (22). Interestingly, we were able to find breakpoints at intron 19 of ALK and intron 6 of CPE at the DNA level in this patient, however, the CPE-ALK start in phase 1 of the first codon of exon 20 may result in the ALK gene being out of frame (**Supplementary Figure S2**). Furthermore, we were unable to guarantee that a fusion is transcribed due to the technical limitations of DNA sequencing. whereas RNA sequencing can accurately identify fusion transcripts, which can supplement fusion detection more effectively (24, 25). Due to insufficient samples, we were unable to perform RNA sequencing. However, the positive expression of ALK IHC and the same expression levels of EML4 and CPE in LUAD and LUSC indicate that CPE-ALK is oncogenic (**Supplementary Figure S1**). This is only a single-patient case report, and more cases are required to investigate the association between ALK fusion and survival benefit in ASC patients. ALK IHC and RNA-NGS (when available) are indispensable complements to DNA NGS for the precise molecular characterization of oncogenic fusions. Additional study and a larger sample size are required to appreciate the biological function of the CPE-ALK or non-classical ALK fusion gene.

The clinical course of ALK-positive NSCLC patients treated with chemotherapy versus ALK-inhibitors differs significantly (26, 27). This observation could be explained by genetic heterogeneity of ALK-positive tumors. The impact of co-mutations on the treatment of ALK-positive patients has been the focus of researchers. TP53, the most prevalent concomitant mutation, has also been shown to be a negative prognostic factor in EGFR mutation NSCLC patients (28). Likewise, ALK-rearranged NSCLC co-occurring TP53 mutations predict an unfavorable outcome of systemic therapy (29), it also tends to suggest that this patient may have a better prognosis.

In conclusion, To the best of our knowledge, this is the first description of a CPE-ALK fusion identified in a patient with ASC who is sensitive to alectinib. this case also expands the spectrum of ALK fusions and provides valuable information on response to alectinib in ASC patients with CPE-ALK fusions, and further investigation is warranted. Overall, Targeted NGS analysis may improve detection of ALK fusions in routine practice.

## Data availability statement

The original contributions presented in the study are included in the article/**Supplementary Material**. Further inquiries can be directed to the corresponding author.

## Author contributions

YQ, FL, YT and QQD prepared the manuscript and the literature search. YQ and FL reviewed and edited the manuscript. YQ treated and observed the patient. FL performed the histopathological, immunohistochemical examinations. All authors contributed to the article and approved the submitted version.

## Acknowledgments

We thank Dr. Chuang Qi, Dr. Wanglong Deng, Dr. Guanghua Lu, Mr. Ran Ding, Mr. Liang Liu, Mr. Binsheng Zhang, and Mr. Liangliang chai from Jiangsu Simcere Diagnostics for their kind assistance.

## Conflict of interest

YT, QD, and QZ are employed by Jiangsu Simcere Diagnostics Co., Ltd. YT, QD, and QZ are employed by Nanjing Simcere Medical Laboratory Science Co., Ltd.

The remaining authors declare that the research was conducted in the absence of any commercial or financial relationships that could be constructed as a potential conflict of interest.

## Publisher’s note

All claims expressed in this article are solely those of the authors and do not necessarily represent those of their affiliated organizations, or those of the publisher, the editors and the reviewers. Any product that may be evaluated in this article, or claim that may be made by its manufacturer, is not guaranteed or endorsed by the publisher.

## Supplementary material

Supplementary Figure 1 CPE and ALK expression level in LUAD and LUSC. A.CPE expression in LUAD and LUSC, T for tumor(red), N for normal(gray); B. EML4 expression in LUAD and LUSC, T for tumor(red), N for normal(gray)

Supplementary Figure 2 Exon 6 information in CPE **(A)** and exon 20 information in ALK **(B)**; Schematic of CPE-ALK may cause the ALK gene to be out of frame **(C)**.

## Abbreviations

ASC, Adenosquamous carcinoma; NSCLC, non-small cell lung cancer; EGFR, epidermal growth factor receptor; ALK, anaplastic lymphoma kinase; PR, partial response; LUAD, lung adenocarcinoma; LUSC, squamous cell carcinoma; TKI, tyrosine kinase inhibitors; CT, Computed tomography; WHO, World Health Organization; IHC, immunochemistry; CEA, carcinoembryonic antigen; CA125, carbohydrate antigen 125.
