# Supplementary figures and images for: Case report: Dramatic response to alectinib in a lung adenosquamous carcinoma patient harbouring a novel CPE-ALK fusion
Source: Front Oncol. 2022 Dec 1;12:998545. doi: 10.3389/fonc.2022.998545 (PMC10111186; doi:10.3389/fonc.2022.998545)

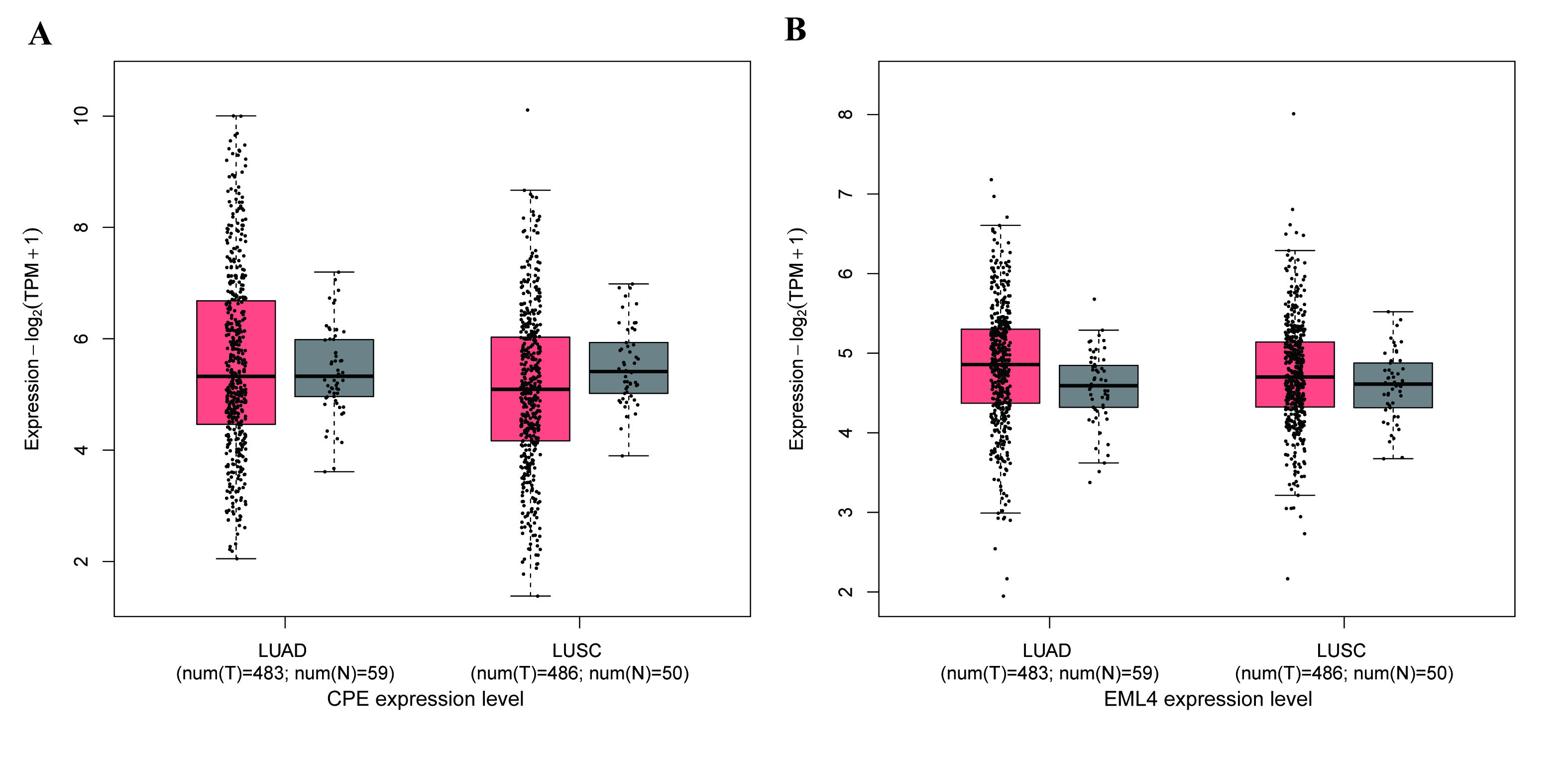

Supplement: Supplementary Figure 1 — CPE and ALK expression level in LUAD and LUSC. A.CPE expression in LUAD and LUSC, T for tumor(red), N for normal(gray); B. EML4 expression in LUAD and LUSC, T for tumor(red), N for normal(gray) [file Image_1.tif]

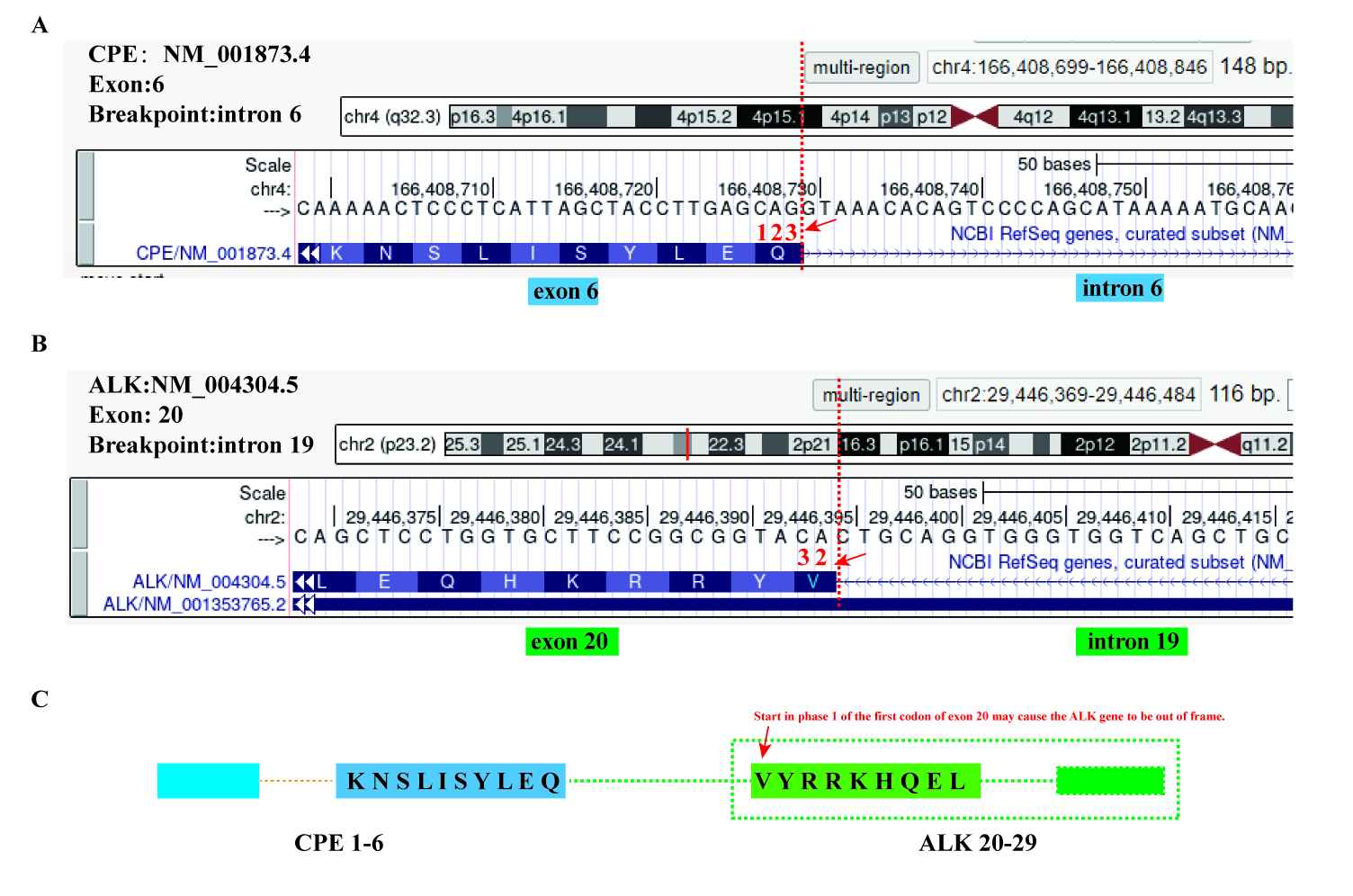

Supplement: Supplementary Figure 2 — Exon 6 information in CPE (A) and exon 20 information in ALK (B); Schematic of CPE-ALK may cause the ALK gene to be out of frame (C). [file Image_2.tif]
