# Supplementary material for: Case report: Dramatic response to alectinib in a lung adenosquamous carcinoma patient harbouring a novel CPE-ALK fusion
Source: Front Oncol. 2022 Dec 1;12:998545. doi: 10.3389/fonc.2022.998545 (PMC10111186; doi:10.3389/fonc.2022.998545)
Supplement: Supplementary file 3 [file Table_1.docx]

| AKT1 | ALK | APC | AR | ARAF | ATM | BCL2L11(BIM) |
| --- | --- | --- | --- | --- | --- | --- |
| BRAF | BRCA1 | BRCA2 | CD274(PD-L1) | CDK4 | CDK6 | CDKN2A |
| CDKN2B | CTNNB1 | DDR2 | EGFR | EPCAM | ERBB2(HER2) | ERBB3 |
| ERBB4 | ESR1 | EWSR1 | FBXW7 | FGFR1 | FGFR2 | FGFR3 |
| FLT3 | GNA11 | GNAQ | HRAS | IDH1 | IDH2 | JAK1 |
| JAK2 | KDR | KIT | KRAS | MAP2K1(MEK1) | MDM2 | MET |
| MLH1 | MPL | MSH2 | MSH6 | MTOR | NF1 | NOTCH1 |
| NRAS | NRG1 | NTRK1 | NTRK2 | NTRK3 | PDCD1(PD-1) | PDGFRA |
| PDGFRB | PIK3CA | PMS2 | POLE | PTEN | RAF1 | RB1 |
| RET | RICTOR | ROS1 | SDHA | SDHB | SDHC | SDHD |
| SMAD4 | SMO | TP53 | TSC2 |  |  |  |

**Supplementary table S1 74 cancer related gene list**
